# Facilitators and barriers for home-based monitoring to time frozen embryo transfers in IVF among women and healthcare providers

**DOI:** 10.1093/hropen/hoac021

**Published:** 2022-05-30

**Authors:** T R Zaat, J P de Bruin, F Mol, M van Wely

**Affiliations:** Department of Obstetrics and Gynaecology, Centre for Reproductive Medicine, Amsterdam Reproduction and Development Research Institute, Amsterdam UMC, University of Amsterdam, Amsterdam, the Netherlands; Department of Obstetrics and Gynaecology, Jeroen Bosch Ziekenhuis, 's-Hertogenbosch, the Netherlands; Department of Obstetrics and Gynaecology, Centre for Reproductive Medicine, Amsterdam Reproduction and Development Research Institute, Amsterdam UMC, University of Amsterdam, Amsterdam, the Netherlands; Department of Obstetrics and Gynaecology, Centre for Reproductive Medicine, Amsterdam Reproduction and Development Research Institute, Amsterdam UMC, University of Amsterdam, Amsterdam, the Netherlands

**Keywords:** assisted reproduction, counselling, embryo transfer, cost-effectiveness, endometrium, menstrual cycle, ART

## Abstract

**STUDY QUESTION:**

What are the facilitators and barriers concerning the implementation of home-based monitoring for natural cycle frozen embryo transfer (NC-FET) from the perspectives of patients and healthcare providers in the Netherlands?

**SUMMARY ANSWER:**

The most important facilitator was optimal pregnancy chance for both the patients and healthcare providers, and the most important barriers were the risk of missing an ovulation for the patients and laboratory capacity for the healthcare providers.

**WHAT IS KNOWN ALREADY:**

The share of FET cycles in IVF treatments is increasing and, therefore, it is important to optimize protocols for FET. Monitoring of ovulation, which is used in NC-FET, can be hospital-based (ultrasounds and ovulation triggering) or home-based (LH urine tests). Home-based monitoring has the advantage of being the most natural protocol for FET and provides the feeling of empowerment and discretion for patients. A systematic approach for the implementation of home-based monitoring has to start with an exploration of the perspectives of all stakeholders.

**STUDY DESIGN, SIZE, DURATION:**

Stakeholders (patients and healthcare providers) involved in the implementation process in the Netherlands participated in the present study. Patients were represented by the Dutch Patient Organisation for Couples with Fertility Problems (FREYA) and healthcare providers were represented by gynaecologists and their society (The Netherlands Society of Obstetrics and Gynaecology), embryologists and their society (The Dutch Federation of Clinical Embryology) as well as fertility doctors. A panel of experts hypothesized on barriers and facilitators for the implementation of home-based monitoring during the proposal phase of the Antarctica-2 randomized controlled trial (RCT).

**PARTICIPANTS/MATERIALS, SETTING, METHODS:**

All stakeholders were represented during the study. Two different questionnaires were developed in order to investigate facilitators and barriers for the patients and for healthcare providers. The facilitators and barriers were ranked on a scale of 1–10 with 10 being the most important. Based on our power analysis, we aimed for a minimum of 300 completed questionnaires for the patients and a minimum of 90 completed questionnaires for the healthcare providers. Facilitators and barriers were analysed using frequencies, mean (SD) and ranking.

**MAIN RESULTS AND THE ROLE OF CHANCE:**

A total of 311 patients filled out the questionnaire of whom 86.8% underwent FET previously. The most important facilitator for the patients was to implement the strategy with the highest chance of pregnancy (mean 9.7; 95% CI 9.6–9.7) and the most important barrier was risk of missing ovulation (mean 8.4; 95% CI 8.2–8.6). A total of 96 healthcare providers filled out the questionnaire. According to healthcare providers, patients would accept the strategy when it causes less interference with their work and private life (mean 7.5; 95% CI 7.1–8.0) and has a low risk of missing the ovulation (mean 7.6; 95% CI 7.1–8.0). The most important facilitator for the implementation of home-based monitoring for healthcare providers was optimizing cumulative pregnancy rates (mean 8.1; 95% CI 7.7–8.4) and the most important barrier was the lack of laboratory capacity and flexibility (mean 6.4; 95% CI 5.8–7.0).

**LIMITATIONS, REASONS FOR CAUTION:**

Facilitators and barriers were selected based on expert opinion. Currently, there are no validated questionnaires that aim to assess facilitators and barriers for the implementation of treatments in fertility care.

**WIDER IMPLICATIONS OF THE FINDINGS:**

During our study, we gained insight into barriers and facilitators for the implementation of home-based monitoring of NC-FET at an early phase. Early sharing and discussion of the results of this study with all stakeholders involved should stimulate a fast incorporation in guidelines, especially as key professionals in guideline development took part in this study. Also, based on our results, we can advise guideline developers to add tools to the guideline that may help overcome the implementation barriers.

**STUDY FUNDING/COMPETING INTERESTS:**

The Antarctica-2 RCT is supported by a grant from the Netherlands Organisation for Health Research and Development (ZonMw 843002807). No authors have any competing interests to declare.

**TRIAL REGISTRATION NUMBER:**

Trial NL6414 (NTR6590).

WHAT DOES THIS MEAN FOR PATIENTS? Frozen-thawed embryo transfer (FET) in the natural menstrual cycle of a woman is based on the timing of ovulation. There are two methods to monitor ovulation: the first is home-based monitoring in which the natural ovulation is monitored using urinary LH tests; the second is hospital-based monitoring using repeated ultrasound monitoring of the largest follicle followed by hCG triggering to induce ovulation. The ongoing Antarctica-2 randomized controlled trial compares the (cost-) effectiveness of home-based monitoring with hospital-based monitoring of the ovulation. If proven to be cost-effective and safe, home-based monitoring can be implemented as the standard care for women with regular menstrual cycles undergoing FET. In the present study, we identified barriers (factors that impede the implementation of, or adherence to, the protocol) and facilitators (factors that promote the implementation of, or adherence to, the protocol) of implementation of home-based monitoring to time FET in the woman’s natural cycle. Patients’ facilitators for home-based monitoring were, in order of importance: a higher pregnancy chance, partner participation and the option to have an ultrasound during the FET. A barrier was risk of missing the ovulation. For healthcare providers, the most important facilitator was a higher pregnancy chance, followed by less transportation inconvenience for the patient, in terms of having to travel at least twice to the fertility centre (for at least one ultrasound and eventually embryo transfer). The most important barrier was the lack of laboratory capacity and flexibility for home-based monitoring. When home-based monitoring is at least as effective as hospital-based monitoring in terms of pregnancy chance, and home urine LH tests do not increase the chance of missing an ovulation, home-based monitoring can easily be implemented from the patient’s point of view.

## Introduction

Frozen embryo transfer (FET) is the second most applied treatment in IVF in Europe, corresponding to annual numbers of 14 257 FET cycles in the Netherlands (LIR, 2019) and 243 302 FET cycles in Europe (Wyns *et al.*, 2021). FET is increasingly successful (Wyns *et al.*, 2021; LIR, 2019) and is enabled by ongoing improvements in laboratory techniques for freezing and thawing of embryos and further boosted by the use of the freeze-all strategy (Wong *et al.*, 2014; Wyns *et al.*, 2021; Zaat *et al.*, 2021).

Although ESHRE recently published a guideline for ovarian stimulation for fresh cycles, no national or international guidelines exist for frozen cycles on how to prepare the endometrium and how to time FET. The recently published Cochrane review on this subject concluded that there is insufficient evidence to recommend one method of endometrial preparation on the basis of pregnancy outcomes in the 31 included randomized controlled trials (RCTs; Glujovsky *et al.*, 2020). Safety issues were not considered in this Cochrane review. FET in the natural cycle (NC-FET) is a safer intervention compared to artificial cycle FET and has been proven to reduce the number of women with hypertensive disorders of pregnancy and placental pathology (Saito *et al.*, 2017, 2019; Ginstrom Ernstad *et al.*, 2019; von Versen-Hoynck *et al.*, 2019; Wang *et al.*, 2020; Zaat *et al.*, 2021). In combination with comparable effectiveness, the interpretation is that NC-FET is the preferred treatment in women with ovulatory cycles undergoing FET when the risks of obstetric complications and potential neonatal complications are considered (Singh *et al.*, 2020; Zaat *et al.*, 2021).

In the Netherlands, two methods for NC-FET are used: hospital-based and home-based monitoring of ovulation. Currently, the ongoing Antarctica-2 RCT with a non-inferiority design is comparing the (cost-) effectiveness of home-based monitoring (LH urine tests) with hospital-based monitoring (ultrasounds and trigger for ovulation) of ovulation to time NC-FET (Zaat, 2021). Whether the use of hCG injection to trigger ovulation in NC-FET is associated with safety issues has not been reported yet. From a biological perspective, we are uncertain whether triggering (i.e. an immature corpus luteum) could impact the safety outcomes after FET. The safety aspect of both home- and hospital-based monitoring will also be assessed in the Antarctica-2 RCT.

If proven to be cost-effective and safe, home-based monitoring can be implemented as the standard care in evidence-based clinical practice guidelines for women with regular menstrual cycles undergoing FET. In this present study, we aimed to identify barriers (factors that impede the implementation of, or adherence to, the guideline) and facilitators (factors that promote the implementation of, or adherence to, the guideline) of implementation of home-based monitoring in order to time FET in the natural cycle. An important factor for the implementation of NC-FET is the possibility and flexibility for both laboratory and clinical planning of FET treatment cycles i.e. the possibility to perform FET on 7 days of the week. Identifying these factors for all stakeholders involved is an important first step for bringing effective interventions into practice (Grol and Grimshaw, 2003; Grol and Wensing, 2004).

## Materials and methods

### Participants

The following stakeholders and organizations in the Netherlands are involved in the implementation process: patients, who are represented by the Dutch Patient Organisation for Couples with Fertility Problems (FREYA); and healthcare providers, represented by gynaecologists and their society (The Netherlands Society of Obstetrics and Gynaecology—special interest group ART), embryologists and their society (The Dutch Federation of Clinical Embryology) and fertility doctors.

Both patients and healthcare providers were asked to fill out a questionnaire. The Medical Ethical Committee (MEC) of the Academic Medical Centre (AMC) reviewed and approved the questionnaires and protocol for the study, and provided a non-WMO (Wet maatschappelijke ondersteuning (Social Support Act)) statement on 29 January 2021 (number 2018_004, MEC AMC, Code 018, https://www.ccmo.nl/metcs/publicaties/publicaties/2018/11/20/erkende-metcs-met-code).

### Selection of facilitators and barriers

According to the framework of Cabana (Cabana *et al.*, 1999) and Fleuren (Fleuren *et al.*, 2004), a panel of experts defined hypothesized barriers and facilitators for the implementation of home-based monitoring during the proposal phase of the Antarctica-2 RCT. The panel of experts defined these barriers and facilitators in seven categories: stimulating patient factors; impeding patient factors; stimulating healthcare provider factors; impeding healthcare provider factors; stimulating health insurance provider factors; stimulating organizational and administrative factors; impeding organizational and administrative factors.

### Assessments

Two different questionnaires were developed in order to investigate facilitators and barriers for patients and healthcare providers separately (Supplementary Data S1 and S2). The questionnaire for healthcare providers also contained questions to investigate the supposed perspective on facilitators and barriers for patients, according to healthcare providers. Furthermore, to gain more insight into the importance of the patient-reported outcomes and patient-reported experiences—assessed in the Antarctica-2 RCT (Zaat *et al.*, 2020)—for healthcare providers, we added two questions to the questionnaire.

The questionnaires were distributed through LimeSurvey (LimeSurvey GmbH, 2003) and were filled out anonymously with only baseline characteristics investigated and not traceable to the patient or healthcare worker. The questionnaire was filled out in the period from March 2021 to November 2021.

### Statistical analysis

To enable differentiation between high- and low-scoring facilitators and barriers with a margin of error of maximally 5%, calculating with a z-score of 1.96, we needed at least 300 patients (population size was set at 1000 for the patients, resulting in a required n = 278) and 90 healthcare providers (population size was set at 110 for the healthcare providers resulting in a required n = 86) to participate in this study.

Facilitators and barriers were ranked on a scale of 1 (not important at all) to 10 (very important) and presented as prescriptive data using frequencies, mean (95% CI) and ranking.

## Results

### Patients

#### Baseline characteristics

A total of 311 patients filled out the questionnaire of whom 86.8% (270/311) underwent FET previously, 24.8% (77/311) in a FET cycle using home-based monitoring. The majority of patients (54.7%) were recruited from IVF centres, i.e. centres that house all clinical and laboratory facilities for IVF/ICSI. The remainder of patients were recruited from so-called transport clinics, which are clinics that co-operate with an IVF centre and perform the stimulation phase, monitoring phase and the oocyte retrieval. The laboratory and embryo transfer phase subsequently take place in the IVF centre (Table I).

**Table I:** Baseline characteristics of patients (n = 311) in a study of facilitators and barriers for home-based monitoring to time frozen embryo transfer in IVF.

Baseline characteristic	% (n)
**Underwent FET**	
Yes	86.8% (270/311)
No	13.2% (41/311)
**Underwent FET in a centre where:**	
The oocyte retrieval, fertilization and embryo transfer took place	54.7% (170/311)
The oocyte retrieval took place, the fertilization and embryo transfer were performed elsewhere	24.8% (77/311)
The monitoring of the FET cycle took place, the oocyte retrieval, fertilization and embryo transfer elsewhere	9.3% (29/311)
No response	9.6% (30/311)
**Type of cycle during FET** *	
Home-based monitoring	24,8% (77/311)
Hospital-based monitoring (serum)	11.3% (35/311)
Hospital-based monitoring (ultrasound)	37.0% (115/311)
Artificial cycle FET	48.6% (151/311)
Other	2.6% (8/311)

* Some of the patients underwent several types of frozen embryo transfer (FET) cycles.

#### Facilitators and barriers for patients for implementing home-based monitoring

**Figure 1.**
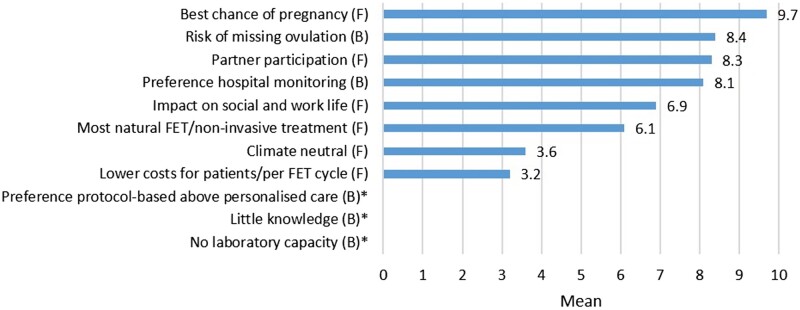
**Facilitators and barriers according to patients.** * was not asked in questionnaire for patients. F, facilitator; B, barrier. The facilitators and barriers are presented as means and ranked on a scale of 1–10 with 10 being the most important.

The facilitators and barriers were ranked on a scale of 1–10 with 10 being the most important. The highest priority for the patients was to implement the strategy with the highest chance of pregnancy (mean 9.7; 95% CI 9.6–9.7) and the lowest risk of missing the ovulation (mean 8.4; 95% CI 8.2–8.6). Furthermore, partner participation (e.g. assistance from the partner with performing urinary LH tests at home) was also ranked as a high priority (mean 8.3; 95% CI 8.1–8.6) followed by the option to have an ultrasound during the FET cycle (mean 8.1; 95% CI 7.8–8.3). Costs for hospital visits were ranked as the least important factor (mean 3.2; 95% CI 2.9–3.5; Table II, Fig. 1).

**Table II:** Facilitators and barriers for patients for implementing home-based monitoring.

	All patients (n = 311)	Patients who had previous FET (n = 270)
**Facilitators**	**Mean (95% CI)**	**Mean (95% CI)**
Highest chance of pregnancy	9.7 (9.6–9.7)	9.7 (9.5–9.8)
Partner participation	8.3 (8.1–8.6)	8.4 (8.1–8.6)
Feeling of empowerment	7.3 (7.0–7.5)	7.4 (7.2–7.6)
Impact on work and social life	6.9 (6.7–7.2)	7.0 (6.8–7.3)
Option for the most natural type of FET cycle	6.1 (5.8–6.4)	6.2 (5.9–6.6)
As climate neutral as possible	3.6 (3.3–3.9)	3.6 (3.3–3.9)
**Barriers**	**Mean (95% CI)**	
Risk of missing ovulation	8.4 (8.2–8.6)	8.5 (8.3–8.7)
Option of an ultrasound	8.1 (7.8–8.3)	8.1 (7.8–8.4)
Visit to the hospital	7.0 (6.8–7.3)	7.1 (6.8–7.4)
Costs for hospital visit	3.2 (2.9–3.5)	3.2 (2.9–3.6)

The importance scale ranged from 1 to 10: 1 = not important at all, 10 = very important.

FET, frozen embryo transfer.

Neither of the two monitoring strategies were clearly preferred by patients (49.8% (151/303) preference for home-based monitoring; 50.2% (152/303) preference for hospital-based monitoring). Most of the patients (94.6% (292/303)) expect to receive personalized care—including several options for type of FET and shared decision-making—and find consultation with their healthcare worker the most helpful way to decide between home- and hospital-based monitoring (79.9% (242/303); Table III). Results of the subgroup of patients who previously completed an FET cycle (86.8%) did not differ compared to the entire group (Table II).

**Table III:** Other factors of importance for implementing home-based monitoring for patients (n = 303*).

Factor	% (n)
**If home-based monitoring is non-inferior to hospital-based monitoring, which type do you prefer?**	
Home-based monitoring	49.8% (151/303)
Hospital-based monitoring	50.2% (152/303)
**What do you expect from your healthcare worker during your FET cycle?**	
Protocol-based care (one option)	3.6% (11/303)
Personalized care (several options, shared decision-making)	96.4% (292/303)
**What do you value as most helpful to make an informed decision between home-based and hospital-based monitoring?**	
Consultation with healthcare worker	79.9% (242/303)
Information folder	6.6% (20/303)
Video with information	7.3% (22/303)
Healthcare workers’ decision	6.3% (19/303)

* Eight patients did not respond to these questions.

FET, frozen embryo transfer.

### Healthcare providers

#### Baseline characteristics

A total of 96 heathcare providers filled out the questionnaire, of whom 64.6% (62/96) were working in a centre with an IVF laboratory. About half of the participants were working as gynaecologists (51% (49/96)). In most cases, hospital-based monitoring using repeated ultrasounds was the standard procedure to time FET in their centres (41.7% (40/96); Table IV).

**Table IV:** Baseline characteristics of healthcare providers (n = 96).

Baseline characteristic	% (n)
**Centre with IVF laboratory**	
Yes	64.6% (62/96)
No	35.4% (34/96)
**Profession**	
Fertility doctor	20.8% (20/96)
Gynaecologist	51.0% (49/96)
Laboratory technician	8.3% (8/96)
Clinical embryologist	18.8% (18/96)
Resident	0.1% (1/96)
Duration of current employment (months, mean (SD))	149.1 (106.5)
**Standard procedure to time FET in centre**	
Home-based monitoring	21.9% (21/96)
Hospital-based monitoring (serum)	2.1% (2/96)
Hospital-based monitoring (ultrasound)	41.6% (40/96)
Artificial cycle FET	20.0% (19/96)
Other	13.5% (13/96)
No response	1.0% (1/96)

FET, frozen embryo transfer.

#### Facilitators and barriers for patients for implementing home-based monitoring and their supposed importance according to healthcare providers

**Figure 2.**
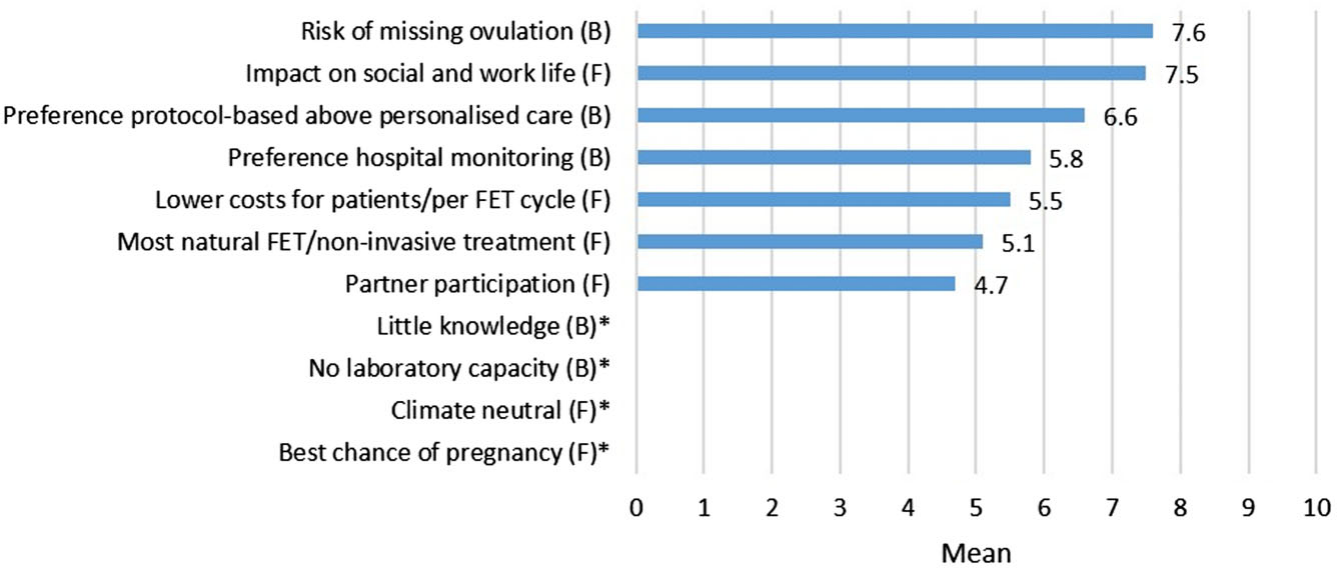
**Facilitators and barriers according: perceptions of the patients according to the healthcare providers.** * was not asked in questionnaire for healthcare providers. F, facilitator; B, barrier. The facilitators and barriers are presented as means and ranked on a scale of 1–10 with 10 being the most important.

Healthcare providers ranked a strong wish for less interference with work and private life (mean 7.5; 95% CI 7.1–8.0) as a dominant facilitator for patients. Healthcare providers ranked a risk of missing the ovulation (mean 7.6; 95% CI 7.1–8.0) as a dominant barrier for patients (Table V, Fig. 2).

**Table V:** Facilitators and barriers for patients for implementing home-based monitoring and their supposed importance according to healthcare providers (n = 96).

Facilitators	Mean (95% CI)
Strong wish for less interference with work and private life	7.5 (7.1–8.0)
Strong wish for personalized care instead of protocol-based care	6.6 (6.1–7.1)
Wish for lower transportation costs	5.5 (5.0–6.1)
Strong wish for non-invasive treatment (e.g. injection)	5.1 (4.6–5.6)
Strong wish for more partner participation during FET cycle	4.7 (4.2–5.3)
Strong wish for no ultrasound monitoring	4.5 (3.9–5.0)

**Barriers**	**Mean (95% CI)**
Risk of missing the ovulation with an LH urine test	7.6 (7.1–8.0)
Preference for monitoring in the hospital by a healthcare worker	5.8 (5.2–6.3)
Preference for artificial cycle FET	4.1 (3.5–4.8)

Score ranging from 1 to 10: 1 = not important at all, 10 = very important.

FET, frozen embryo transfer.

#### Facilitators and barriers for healthcare providers for implementing home-based monitoring

**Figure 3.**
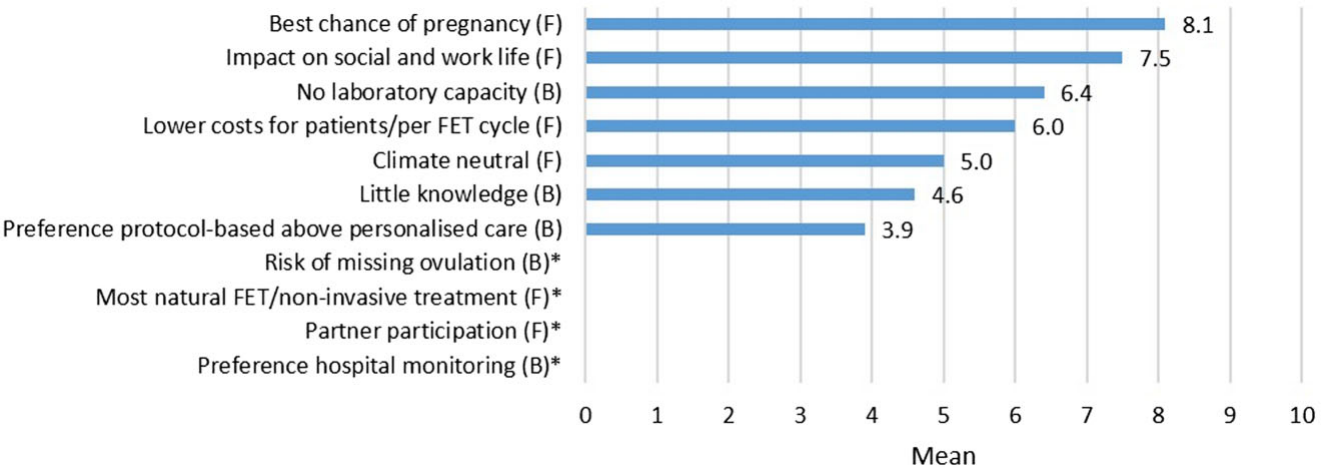
**Facilitators and barriers according to healthcare providers.** * was not asked in questionnaire for healthcare providers. F, facilitator; B, barrier. The facilitators and barriers are presented as means and ranked on a scale of 1–10 with 10 being the most important.

The most important facilitator for healthcare providers was optimizing cumulative pregnancy rates (mean 8.1; 95% CI 7.7–8.4) and the most important barrier for implementation of home-based monitoring for healthcare providers was the lack of laboratory capacity and flexibility (mean 6.4; 95% CI 5.8–7.0; Table VI, Fig. 3).

**Table VI:** Facilitators and barriers for healthcare providers from implementing home-based monitoring (n = 96).

Facilitators	Mean (95% CI)
Optimizing the cumulative pregnancy rates	8.1 (7.7–8.4)
Less transportation inconvenience for the patient/less interference with work and private life	7.5 (7.1–7.9)
Lower costs per FET cycle	6.0 (5.4–6.6)
As climate neutral as possible	5.0 (4.3–5.8)

**Barriers**	**Mean (95% CI)**
No laboratory capacity and flexibility for home-based monitoring	6.4 (5.8–7.0)
Less control on timing of FET and therefore maybe more FET during weekends	5.6 (4.9–6.3)
Little to no knowledge about the efficacy of home-based monitoring	4.6 (4.0–5.2)
Preference for protocol-based care above personalized care	3.9 (3.3–4.4)

Score ranging from 1 to 10: 1 = not important at all, 10 = very important.

FET, frozen embryo transfer.

The majority of healthcare providers (79.0% (49/62)) indicated that from a patients’ perspective, empowerment and discretion facilitate home-based monitoring. Most of the healthcare providers (79.0% (49/62)) expected that if home-based monitoring turns out to be non-inferior to hospital-based monitoring, home-based monitoring will be implemented in their centre. Of the 13 healthcare providers who did not expect home-based monitoring to be implemented in their centre, the most common reasons were the expectation that patients prefer hospital-based monitoring, and logistic problems for the IVF laboratory (Table VII).

**Table VII:** Other factors of importance for healthcare providers for implementing home-based monitoring (n = 62*).

Factor	% (n)
**Do you expect the fact that women feel more ‘empowered’ and experience the treatment as more discrete during home-based monitoring to have a motivating effect on implementation of home-based monitoring in your centre?**	
Yes	79.0% (49/62)
No	21.0% (13/62)
**If no, the reason home-based monitoring will probably not be implemented in my centre is:**	
Logistic problems for the outpatient clinic	7.7% (1/13)
Logistic problems for the IVF laboratory	38.5% (5/13)
Patients’ preference: hospital-monitored	46.2% (6/13)
Financial concerns	0.0% (0/13)
Expert convictions resistant to change	0.0% (0/13)
Lack of knowledge about changes	15.4% (2/13)
**If home-based monitoring is non-inferior to hospital-based monitoring based on the results of the ANTARCTICA-2 study, do you expect this study-result to have a motivating effect on implementation of home-based monitoring in your centre?**	
Yes	79.0% (49/62)
No	21.0% (13/62)
**If no, the reason home-based monitoring will probably not be implemented in my centre is:**	
Logistic problems for the outpatient clinic	15.4% (2/13)
Logistic problems for the IVF laboratory	46.2% (6/13)
Patients’ preference: hospital-monitored	53.8% (7/13)
Financial concerns	7.7% (1/13)
Expert convictions resistant to change	15.4% (2/13)
Lack of knowledge about changes	15.4% (2/13)

* Thirty-four healthcare providers did not respond to these questions.

FET, frozen embryo transfer.

We ranked the following factors for successful implementation of home-based monitoring from most important to least important, according to healthcare providers: update of current guidelines (38.7% (24/62)); presenting study results in the IVF centres in the Netherlands (33.9% (21/62)); presenting study results at international conferences (14.5% (9/62)); information on website of Dutch Patient Organisation (11.3% (7/62)), master version for local protocol composed by the Antarctica-2 study group (1.6% (1/62); Table VIII).

**Table VIII:** Factors of importance for successful implementing home-based monitoring according to healthcare providers (n = 62*).

Factor	Rank 1	Rank 2	Rank 3	Rank 4	Rank 5
Update of current guidelines	38.7% (24/62)	27.9% (17/61)	11.7% (7/60)	13.3% (8/60)	10.0% (6/60)
Presenting study results at international conferences	14.5% (9/62)	24.6% (15/61)	15.0% (9/60)	15.0% (9/60)	30.0% (18/60)
Presenting study results in the IVF centres in the Netherlands	33.9% (21/62)	24.6% (15/61)	21.7% (13/60)	15.0% (9/60)	5.0% (3/60)
Master version for local protocol composed by the ANTARCTICA-2 study group	1.6% (1/62)	16.4% (10/61)	35.0% (21/60)	26.7% (16/90)	20.0% (12/60)
Information on website of Dutch Patient Organisation	11.3% (7/62)	6.6% (4/61)	16.7% (10/60)	30.0% (18/60)	35.0% (21/60)

Ranking 1 to 5: 1 = most important, 5 = least important.

*Thirty-four healthcare providers did not respond to these questions.

## Discussion

In this study, we aimed to identify the facilitators and barriers for the implementation of home-based monitoring for FET in the natural cycle, as seen from perspectives of patients and healthcare providers in the Netherlands.

Patients’ facilitators for home-based monitoring were in order of importance: the best chance of pregnancy, partner participation and the option to have an ultrasound during the FET. A barrier was the risk of missing the ovulation. The healthcare workers generally had a good idea of the facilitators and barriers for patients. For healthcare providers, the most important facilitator was a higher pregnancy chance, followed by less transportation inconvenience for the patient. The most important barrier was the lack of laboratory capacity and flexibility for home-based monitoring.

Overall, facilitators and barriers for patients for implementing home-based monitoring and their supposed importance for patients according to healthcare providers were comparable.

Previously performed studies showed that home-based ultrasound monitoring of follicle growth in fresh IVF cycles improved patient-reported outcomes and experiences such as empowerment and discretion. These findings were comparable to the results of the patient-reported outcomes and experiences study in the first part of the Antarctica-2 RCT (Zaat *et al.*, 2020). Therefore, when home-based monitoring is at least as effective as hospital-based monitoring in terms of pregnancy chance, and home urine LH tests do not increase the chance of missing an ovulation, home-based monitoring can easily be implemented from the patient’s point of view.

For healthcare providers, the most important barrier for the implementation of home-based monitoring is the lack of laboratory capacity and flexibility for home-based monitoring. The lack of laboratory capacity for thawing or warming of embryos, especially at weekends, as a barrier in the Netherlands, has been derived from the expert panel. The Antarctica RCT assessed the impact on the weekend days when using hospital-based monitoring in NC-FET. The results showed that only one in every seven FET was scheduled on a weekend day (Groenewoud *et al.*, 2016). The exact timing of FET in the natural cycle is still unclear based on the current literature. We hypothesize that timing is somewhat flexible considering the ‘window’ of ovulation in the natural cycle. This hypothesis needs further investigation.

To overcome previously mentioned problems regarding the implementation of NC-FET, first, the logistics of the laboratory need to accommodate the most effective, safe and patient preferred strategy for FET. Second, according to the healthcare providers, successful implementation of home-based monitoring will be achieved by updating the current guidelines. Presenting study results at national and international level is also rated to be highly important for implementation. Other suggestions made by healthcare providers for successful implementation focused mainly on ways to increase the weekend capacity by hiring more employees and financial budgets to do so. Furthermore, suggestions were made on the availability of reliable and easy to use LH tests, which are covered by insurance.

A major strength of this study is the involvement of all stakeholders—including key professionals in guideline development—in the implementation process. We used a 10-point scale, which results in a reliable and fine-grained assessment of the importance of each facilitator and barrier.

To our knowledge, this study is the first to report on facilitators and barriers in the implementation process of NC-FET. Considering the increasing number of FET cycles worldwide, it is of great importance to gain more knowledge about factors of importance during the implementation of NC-FET. It should be noted that the Antarctica-2 RCT is still ongoing. Early sharing and discussion of facilitators and barriers found will help to optimize adequate and in-time implementation of the eventual study results.

The outcomes of this study are generalizable to Dutch patients and healthcare providers. However, it should be noted that a small number (16.3%) of the participating patients had never undergone FET and filled out the questionnaire based on hypothesized opinion rather than personal experience.

With the Netherlands being a densely populated country, we do wonder whether the results of our study are generalizable, especially when looking at less populated or sparsely populated countries. Home-based monitoring may be very useful in that case but, in relation to the repeated hospital visits for ultrasound monitoring hospital-based monitoring is probably not.

Facilitators and barriers were selected based on expert opinion. Currently, there are no validated questionnaires that aim to assess facilitators and barriers for the implementation of treatments in fertility care. This uncertainty should be acknowledged. Concerning implications for future research, the development of validated assessment tools to identify facilitators and barriers during different processes in fertility care is preferable. It would be interesting to perform a second questionnaire after implementation of home-based monitoring FET in order to gain more insight into the process itself for future reference in facilitator and barrier studies. In the questionnaires for both patients and for healthcare providers regarding patient perceptions, we investigated 11 topics for facilitators and barriers. Based on our previously published study on patient-reported outcomes and experiences (Zaat *et al.*, 2020), we concluded that the factors of empowerment and discretion are important and already known to affect patients’ perceptions.

Evidence-based clinical practice guidelines are intended to assist with patient and healthcare worker decisions about appropriate healthcare for specific clinical circumstances. However, studies in countries such as the USA and the Netherlands have suggested that at least 30–40% of patients do not receive care according to current scientific evidence (Bosse *et al.*, 2006). Translating evidence from guidelines into practice, also known as implementation, is a challenging process as it involves making changes at the individual, organizational or health system levels (Rabin *et al.*, 2008; Litjens *et al.*, 2013). Knowledge about facilitators and barriers for all stakeholders involved is an important first step in the challenging process of implementation of effective interventions into practice (Grol and Grimshaw, 2003; Grol and Wensing, 2004; Rabin *et al.*, 2008; Litjens *et al.*, 2013).

During our study, we gained insight into barriers and facilitators for the implementation of home-based monitoring of NC-FET at an early phase in order to advise the guideline development group in adding tools to the guideline to help overcome the barriers identified. From a historic point of view, efficacy is the main focus as an outcome measure in clinical research and guideline development. Quiet recently, safety outcomes were valued as being equally important. For future guideline development, we recommend to only include safety and efficacy outcomes as the primary guiding principles. Patient preference is difficult to include in this balance but should be taken into account as the secondary focus in guideline development (Elf *et al.*, 2017; Gentry and Badrinath, 2017). Early sharing and discussion of the results of this study with all stakeholders involved should stimulate fast incorporation into guidelines, especially as key professionals in guideline development took part in this study. Also, based on our results, we can advise guideline developers to add tools to the guideline that may help overcome implementation barriers.

In conclusion, concerning the implementation process, the study results show that the most important facilitator was optimal chance of pregnancy, and the most important barriers were risk of missing an ovulation and laboratory capacity. Based on current research, NC-FET is as effective as artificial cycle FET (Glujovsky *et al.*, 2020) and is probably safer in terms of pregnancy and child outcomes (Saito *et al.*, 2017, 2019; Ginstrom Ernstad *et al.*, 2019; von Versen-Hoynck *et al.*, 2019; Wang *et al.*, 2020; Zaat *et al.*, 2021). Before a final recommendation regarding NC-FET using home-based or hospital-based monitoring can be made, we need to await completion of the Antarctica-2 RCT, which will generate data on pregnancy rates, cost-effectiveness and safety (Zaat, 2021). In our opinion, the logistics of the laboratory need to accommodate the most effective and safest strategy for FET.

## Data availability

The data underlying this article will be shared on reasonable request to the corresponding author.

## Supplementary Material

hoac021_Supplementary_Data1

hoac021_Supplementary_Data2
