# Supplementary material for: Facilitators and barriers for home-based monitoring to time frozen embryo transfers in IVF among women and healthcare providers
Source: Hum Reprod Open. 2022 May 30;2022(3):hoac021. doi: 10.1093/hropen/hoac021 (PMC9188296; doi:10.1093/hropen/hoac021)
Supplement: hoac021_Supplementary_Data1 [file hoac021_supplementary_data1.docx]

**Supplementary Data 1**

**Questionnaire: Facilitators and Barriers for patients implementing home-based monitoring**

Currently the ANTARCTICA-2 study is an ongoing trial comparing the (cost-)effectiveness of home-based monitoring of the ovulation with hospital-based monitoring of the ovulation in order to time FET in IVF. This study is conducted in 22 sites in the Netherlands and is now in the last phase of inclusion. With this questionnaire we want to gain more insight in the implementation process of the use of home-based monitoring. We highly appreciate your help!

**Facilitators and barriers for patients**

Implementation of home-based monitoring may result in the following facilitators and barriers for patients. How important do you rate the following factors on a scale of 1 (not important at all) to 10 (highly important) during FET in the natural cycle?

Factor 1: How important do you value the risk of missing the ovulation during your FET-cycle?

Factor 2: How important do you value the option of an ultrasound during your FET-cycle?

Factor 3: How important do you value partner participation during your FET-cycle?

Factor 4: How important do you value the feeling of empowerment during your FET-cycle?

Factor 5: How important do you value the impact on your social life of your FET-cycle?

Factor 6: How important do you value the impact on your professional life of your FET-cycle?

Factor 7: How important do you value a visit to the hospital during your FET-cycle?

Factor 8: How important do you value the option of the most natural type of FET-cycle?

Factor 9: How important do you value the highest chance of pregnancy during your FET-cycle?

Factor 10: How important do you value the costs you have to make for a hospital visit during your FET-cycle?

Factor 11: How important do you value the treatment to be as climate-neutral as possible?

**Other factors of importance for implementing home-based monitoring**If home-based monitoring results in the same changes of pregnancy compared to hospital-based monitoring, what would you prefer:

Home-based monitoring of the ovulation
Hospital-based monitoring of the ovulation

What do you expect from your healthcare worker during your FET cycle?

Your healthcare worker will treat you according to protocol-based care: the choice will be made for you based on current protocols
Your healthcare worker will treat you based on personalised care: the choice will be made using shared decision making with you

What do you value most helpful to make an informed decision between home-based monitoring and hospital-based monitoring?

Consultation with your healthcare worker discussion your options for the FET-cycle
Reading an information folder about your options for the FET-cycle
Watching a video about your options for the FET-cycle
I would prefer that my healthcare worker decides for m

Suppose that a certain protocol for FET has an increased risk of developing pregnancy complications such as hypertensive disorder of pregnancy, will this change your decision?

Yes
No
