# Supplementary material for: Facilitators and barriers for home-based monitoring to time frozen embryo transfers in IVF among women and healthcare providers
Source: Hum Reprod Open. 2022 May 30;2022(3):hoac021. doi: 10.1093/hropen/hoac021 (PMC9188296; doi:10.1093/hropen/hoac021)
Supplement: hoac021_Supplementary_Data2 [file hoac021_supplementary_data2.docx]

**Supplementary Data 2**

**Questionnaire: Facilitators and Barriers for health care providers for implementing home-based monitoring**

Currently the ANTARCTICA-2 study is an ongoing trial comparing the (cost-)effectiveness of home-based monitoring of the ovulation with hospital-based monitoring of the ovulation in order to time FET in IVF. This study is conducted in 22 sites in the Netherlands and is now in the last phase of inclusion. With this questionnaire we want to gain more insight in the implementation process of the use of home-based monitoring. We highly appreciate your help!

**Facilitators for patients**
Implementation of home-based monitoring may result in the following facilitators for patients. How important do you rate the facilitators on a scale of 1 (not important at all) to 10 (highly important)?

Strong wish for personalised care instead of protocol-based care

Strong wish for more partner participation during FET cycle

Strong wish for less interference with work and private life
Wish for lower transportation costs
Strong wish for no ultrasound monitoring
Strong wish for non-invasive treatment (e.g. injection)

**Barriers for patients**
Implementation of home-based monitoring may result in the following barriers for patients. How important do you rate the barriers on a scale of 1 (not important at all) to 10 (highly important)?

Risk of missing the ovulation with LH urine test
Preference for monitoring in the hospital by a healthcare worker
Preference for artificial cycle FET

**Facilitators for healthcare workers**
Implementation of home-based monitoring may result in the following facilitators for healthcare workers. How important do you rate the facilitators on a scale of 1 (not important at all) to 10 (highly important)?

Optimising the cumulative pregnancy rates
Lower costs per FET cycle
Less transportation inconvenience for the patient
As climate-neutral as possible

**Barriers for healthcare workers**
Implementation of home-based monitoring may result in the following barriers for healthcare workers. How important do you rate the barriers on a scale of 1 (not important at all) to 10 (highly important)?

No laboratory capacity and flexibility for home-based monitoring
Less control on timing of FET and therefore maybe more FET during weekends
Little to no knowledge about the efficacy of home-based monitoring
Preference for protocol-based care above personalised care

**Other factors of importance for implementing home-based monitoring**Based on the subgroup analysis of the ANTARCTICA-2 study concerning ‘*patient reported outcomes and experiences’* women experience more ‘empowerment’ and more discretion during home-based monitoring compared to hospital-based monitoring. Do you expect these study-results to have a motivating effect on implementation of home-based monitoring in your centre?

Yes

No

🡪 If no, the reason home-based monitoring will probably not be implemented in my centre is:

Logistic problems for the outpatient clinic (e.g. weekends)

Logistic problems for the IVF laboratory(e.g. weekends)

Patients’ preference to be monitored in the hospital by a health care worker

Financial concerns

Expert convictions resistant to change

Lack of knowledge about the study results or changes in national guidelines

**Ref: Zaat 2020*

**I**f home-based monitoring is non-inferior to hospital-based monitoring based on the results of the ANTARCTICA-2 study, do you expect this study-result to have a motivating effect on implementation of home-based monitoring in your centre?

Yes

No

🡪 If no, the reason home-based monitoring will probably not be implemented in my centre is:

Logistic problems for the outpatient clinic (e.g. weekends)

Logistic problems for the IVF laboratory(e.g. weekends)

Patients’ preference to be monitored in the hospital by a health care worker

Financial concerns

Expert convictions resistant to change

Lack of knowledge about the study results or changes in national guidelines

What factors are necessary for optimal implementation of the results of the ANTARCTICA-2 study according to you? Please sort the factors as stated below in order of importance (first being the most important and sixth being the least important).

Update of current guidelines

Presenting study results at international conferences/meetings

Presenting study results in the IVF-centres in the Netherlands (for example by pre-recorded video)

Master version for local protocol composed by the ANTARCTICA-2 study group

Information published on the website of the Dutch Patient Organisation for Couples with Fertility Problems

Other, elaborate:
